# Mindfulness and mental health: the importance of a clinical intervention to prevent the effects of a traumatic event. A pilot study

**DOI:** 10.3389/fpsyg.2024.1449629

**Published:** 2024-10-22

**Authors:** Cristina Liviana Caldiroli, Rossella Procaccia, Attà Negri, Andrea Mangiatordi, Silvia Sarandacchi, Alessandro Antonietti, Marco Castiglioni

**Affiliations:** ^1^Department of Human Sciences “Riccardo Massa”, University of Milano-Bicocca, Milan, Italy; ^2^Faculty of Psychology, eCampus University, Novedrate, Italy; ^3^Department of Human and Social Sciences, University of Bergamo, Bergamo, Italy; ^4^Department of Psychology, Catholic University of the Sacred Heart, Milan, Italy

**Keywords:** mindfulness, positive psychology, wellbeing, COVID-19, pandemic, trauma, clinical intervention

## Abstract

Numerous research studies show that mindfulness can mitigate the negative impact of trauma on mental health by reducing symptoms of anxiety, mediating the relationship between trauma exposure and mental health, and treating symptoms resulting from traumatic events. During the COVID-19 pandemic, which was considered a traumatic event, the wellbeing of adults and children was severely compromised. Although children seem less vulnerable to the physical effects of the virus, this does not seem to be true for the psychological effects. Indeed, a prolonged period of loss of family activities and routines can have a negative impact on the mental health of children and adolescents. To investigate how mindfulness can help preschool children cope with the effects of COVID-19, a study was conducted on 46 children aged 4–5 years. The programme, based on the work of Jon Kabat-Zinn and adapted to the age of the participants, consisted of eight weekly 45-min sessions. Qualitative and quantitative results showed positive feedback, indicating that mindfulness helps children make sense of their experiences and achieve functional post-traumatic growth. This approach is seen as a challenge to guide children toward the restoration of psychological wellbeing, which is essential for good psychological balance.

## 1 Introduction

Mindfulness, in its original etymology, refers to a state of mental presence in which one sees internal phenomena as they really are and discriminates between external phenomena and one's own mental projections and distortions (Rinpoche, 1998; Uchiyama, 2004). According to the original tradition, mainly based on Buddhist meditation teachings, mindfulness practice should allow one to move from a state of disequilibrium and suffering to one of greater subjective wellbeing, thanks to a deep knowledge of one's mental states and processes (Shapiro et al., 2006). Since the 1960s, there has also been an interest in meditative practices within clinical psychology, where there has been recognition of the potential for the development of awareness in patients, with important implications for their wellbeing (Keng et al., 2011). The contribution of the Zen Buddhist monk Hanh (1976) and the development of his teachings in the context of clinical and experimental psychology by Kabat-Zinn (1990) were crucial to the cultural integration between Buddhist meditative practice and the new Western forms of mindfulness.

Mindfulness is characterized by two closely related components: the ability to bring attention to the present moment (intentionality, attentional self-regulation) and the attitude of doing so, consisting of curiosity, opennes, and acceptance (Bishop et al., 2004). These components allow the person to relate to his or her experiences in a mindful way, with the attitude of being present to them, but in a non-critical and non-anxious way. This metacognitive awareness corresponds to a decentralized and disarmed way of relating to our thoughts, emotions, and sensations in the present, where the person experiences mental states as mental states rather than as objective reality (“thought is not fact, nor am I”). In this way, mindfulness is seen as a specific way of experiencing the inner and outer world with conscious attention. Without judging them or identifying with their mental content, we accept them moment by moment as they are.

The Mindfulness Based Stress Reduction (MBSR) programme was formulated by Kabat-Zinn in the 1970s. This was the first structured mindfulness-based intervention presented to the scientific community as an effective treatment for pain (Kabat-Zinn, 1982). Since then, there has been a proliferation of research on mindfulness practices and their effects in different populations and settings. This has led to the emergence of many mindfulness-oriented clinical practices, such as Dialectical Behavioral Therapy (Linehan, 1993), Acceptance and Commitment Therapy (Hayes et al., 1999), and Mindfulness-Based Cognitive Therapy (Segal et al., 2002). The efficacy of mindfulness as a therapeutic approach for adults has been demonstrated in numerous clinical conditions, both organic (chronic pain, rheumatoid arthritis, fibromyalgia) and psychological (reducing stress in cancer patients, anxiety, mood disorders, substance abuse) (e.g., Segal et al., 2002; Mace, 2008). More recently, there was the need to implement interventions that help the younger population to restore their psychophysical balance and their individual and social wellbeing (Behan, 2020; Courtney et al., 2020; Xie et al., 2020; Andreu et al., 2023), building on the existing literature on effective interventions with children and adolescents in the case of traumatic and stressful events and replicating them in the current context (Rempel, 2012; Follette et al., 2006; Romeo, 2017; Caldiroli et al., 2020; Kachadourian et al., 2021; Castiglioni et al., 2022; Garcia-Rubio et al., 2023). In particular, there has been a growing interest in the theoretical research and clinical applications of mindfulness in childhood (Greenberg and Harris, 2011; Fabbro and Muratori, 2012; Caldiroli et al., 2020) and, in particularly in school context, working as a preventive method to stress, anxiety, negative emotions, lack of attention or self-regulation (Bockmann and Yu, 2023).

Since mindfulness-based interventions can be useful in coping with stressful situations by promoting self-directed attention, teaching acceptance of negative thoughts and feelings, and promoting non-judgement (Kabat-Zinn, 1994; Goodman and Schorling, 2012; Weis et al., 2021) the present pilot study aims to investigate the protective and preventive value of mindfulness by focusing on one of the most recent events that has had a strong impact on the mental and physical wellbeing of the entire population: the COVID-19 pandemic. The pandemic outbreak in Wuhan in December 2019 had a significant impact on the entire population, drastically altering daily routines and severely affecting individuals' physical and mental health (e.g., Brooks et al., 2020; Fiorillo and Gorwood, 2020; Rudnik et al., 2021; Miyah et al., 2022; Castiglioni et al., 2023; Negri et al., 2023). As with the adult population, children and adolescents also experienced and faced an unprecedented event, with profound alterations in their lifestyles that interfered with their social development in a way that could have long-term ramifications. Faced with this situation and an uncertain future, new worries and anxieties were identified in children. There was a worsening of depressive symptoms, an increase in behavioral problems and social withdrawal, a decrease in self-esteem, and an increase in anxiety and stress (Pfefferbaum and North, 2020; Gonda and Tarazi, 2022).

Mindfulness-based protocols have been found to be effective in the promotion of wellbeing and adjustment in school populations (Beauchemin et al., 2008; Saltzman and Goldin, 2008; Duff, 2024) and in individuals with mental disorders (Biegel et al., 2009; McKeen et al., 2023). In particular, studies have shown that mindfulness-based therapeutic interventions show positive results in the treatment of children and adolescents with oppositional defiant disorder, conduct disorder, attention deficit hyperactivity disorder (ADHD), anxiety, depression, obsessive-compulsive disorder, chronic pain, and substance abuse (Burke, 2010; Zenner et al., 2014; Kallapiran et al., 2015; Swain et al., 2015; Tan, 2016; Zoogman et al., 2015; Black and Slavich, 2016; Cairncross and Miller, 2020; Felver et al., 2016; Evans et al., 2018; Carsley et al., 2018; Caldiroli et al., 2020, 2021). With mindfulness, children and young people increase awareness (react less automatically) and learn to be “consciously present” in the breath, body (tastes, smells, images, sounds), thoughts, emotions, needs, actions, others, and environment (Baer et al., 2006; Chiesa et al., 2013). Mindfulness in developmental age is considered a promising therapeutic practice (Fabbro and Muratori, 2012).

In the current research it was decided to use a mindfulness protocol adapted by Montano and Villani (2018) from the MBSR programme with kindergarten children. The main focus of the present study is to investigate the possible impact of mindfulness on cognitive, emotional, expressive, behavioral and awareness aspects of the individual and, in particular, on attention, memory, creativity, emotional intelligence, interpersonal relationships, awareness, and behavioral control. The aim is to assess the possible benefits and improvements that the training can have on stress management and regulation of thoughts and feelings, especially in a period such as the last few years.

## 2 Materials and methods

### 2.1 Participants

A total of 46 children aged between 4 and 5 years participated in the study. The sample consisted of 22 children (10 boys, 12 girls) in the experimental group and 24 children (11 boys, 13 girls) in the control group. The children were divided into the two groups according to the school they attended. It should be noted that at a later stage, at the end of the experimental phase, mindfulness training was also given to the control group. Parents and teachers collaborated in the research to complete the questionnaires and tests.

### 2.2 Procedure and measures

Several schools in northern Italy were asked to participate in the study by an invitation letter. The first three schools to reply to the invitation were selected for the present pilot study. A quasi-experimental research design with non-equivalent groups and only post-tests was set up. In this research design, the participants are not randomly assigned to the experimental and control groups; consequently, from a methodological point of view, the researcher only compares situations that have already been predetermined in the natural context (Fiorillo and Gorwood, 2020; Cook et al., 1990; Gopalan et al., 2020; Trinchero, 2004).

The pilot study took place from January to December 2022 in three kindergartens on a voluntary basis. During this period, meetings were organized with the school and parents to present the programme and explain its objectives, training sessions with the children took place, and, at the end of the programme, a feedback meeting involved teachers and parents.

Several instruments were used to evaluate the effects of mindfulness training on children, in particular: parents were somministred with two pre-training questionnaires and one post-training questionnaire to assess the effects of COVID-19 on children's lives and the impact of mindfulness training. In addition, a pre-post training questionnaire was administered to assess the children's skills. In the pre-training phase, the questionnaire was completed by the teacher before starting the mindfulness programme. In the post-training phase, the questionnaire was completed by the teacher at the end of the mindfulness programme.

The sessions with the children took place in a noise-free room and with minimal distractions. The 4- and 5-year-old children were divided into two groups on the same day, maintaining the same protocol, room, and experimenter for both groups. Each session began with the placement of mats on the floor, providing the children with a sense of personal space and a secure base. The control group completed the questionnaires at the same time as the experimental group, but without participating in the training. Subsequently, the control group was also administered the mindfulness protocol. To evaluate the effects of the mindfulness training on the children, various instruments were used, including specific questionnaires to investigate any psychological problems and discomforts that had arisen due to the COVID-19 pandemic and to assess benefits and improvements resulting from the mindfulness programme.

#### 2.2.1 Modified and adapted mindfulness programme

Based on the general protocol of Montano and Villani (2018), a simplified protocol was developed, suitable for 4- and 5-year-old children. The features that remained unchanged from the original protocol are: 8 sessions; from 45 to 60 min of duration per session; the weekly schedule; the themes and the order of the sessions. The first session is an introduction to mindfulness and personal “secret garden”; the second is based on the concept of the beginner's mind; the third focuses on thinking and the “wandering mind”; the fourth is based on emotions and emotional intelligence; the fifth concentrates on dealing with stressful events and non-judgment; the sixth is about awareness before action; the seventh session introduces awareness of self and others as a support for difficult communication; and finally the eighth session consolidates attitudes of kindness toward oneself and concludes the programme.

However, to adapt to the needs of younger children, some aspects had to be modified: the number of exercises per session was reduced: one for initial meditation, one in the middle, and one at the end; the time for reviewing the tasks was retained, but an attempt was made to limit the time to a maximum of 15 min (with discussion led by the experimenter); only one psycho-educational moment was established, characterized by one of the proposed activities, which was analyzed in depth with the experimenter; only one reading moment was maintained, with the selection of the passages indicated for the younger children, which were carefully analyzed and contextualized during the group discussion (also guided by the experimenter). It turned out that the children were able to follow and remained attentive and interested throughout the sessions, thanks to the way they were set up. The sessions were structured into three phases as described in Table 1.

**Table 1:** General structure of meetings.

**Initial phase**	**Middle phase**	**Final phase**
**Formal practice:** Opening meditation based on breathing and self-awareness	**Formal practice:** Central meditation based on body awareness and non-judgement	**Formal practice:** Closing meditation to bring them back to the here and now
**Review of homework:** Time to share the efforts and positive aspects experienced during the proposed activities	**Reading moment:** Texts based on the main theme of the session	**Homework delivery:** Home activities based on the main theme of the session
**Psychoeducational moments:** Didactic presentations given by the teachers	**Group discussion:** Sharing experiences related to the main theme	

^*^For a complete overview of the programme, please refer to the book “Il fiore dentro” (Montano and Villani, 2018).

#### 2.2.2 Pre-training questionnaire - COVID-19 effects

Parents were asked to respond regarding their child over the past 12 months. The questionnaire consists of a first part of open-ended questions and a second part where they are asked to give their opinion on a scale of 1 (not at all) to 5 (very much). The open questions collected personal data (e.g., age; family composition; who the child lives with; etc.) and data about the period of the pandemic (e.g., whether the child had COVID-19; whether any family members or acquaintances had it; whether the child's school was closed because of COVID-19 and, if so, how often it was closed; whether the child engages in recreational and/or sports activities after school and how they were managed during the pandemic; etc.). The closed questions were more concerned with children's feelings and needs (e.g., did the child feel sorry for the school closure; did the child show more attachment to caregivers since the pandemic; did the child show more irritability/angry since the pandemic; did the child feel more need to do outdoor activities since the pandemic; etc.).

#### 2.2.3 Pre/post parent questionnaire—Mindfulness

*Ad hoc* questionnaire to assess mindfulness programme impact. In particular, it looks at how the child relates to, manages, recognizes, and expresses emotions, thoughts and feelings about themselves and others before and after the training. The questionnaire was given to the parents. They were asked to rate their child on a scale from 1 (not at all) to 5 (very much). For example, it was asked whether the child shows irritability, whether he is a restless sleeper, whether he is an attentive child, whether he is a creative child, whether he is curious, whether he is able to regulate his emotional reactions (such as fear, joy, sadness), whether he knows how to recognize mistakes, whether he listens to instructions before starting a task, etc.

#### 2.2.4 Questionnaire for the assessment of skills in children—SAI (pre/post training)

This is a tool proposed by A. Antonietti, R. Finazzi and M. Geroldi of the Service of Psychology of Learning and Education in the Age of Development (SPAEE) of the Catholic University of the Sacred Heart. It is designed to assess the abilities (skills) of children around the age of 5. The instrument evaluates a wide range of skills, each with a similar section: attention, memory, spatial cognition, reasoning, creativity, language, feelings, interpersonal relations, motor skills, awareness, planning, and behavioral control. The questionnaire is made up of 24 items, each with 4 possible answers, and the teacher must choose the one that best fits the child. Each skill is presented in relation to a specific situation, so that the respondent can think of a concrete case in which the skill is manifested. In the pre-training phase, the questionnaire was completed by the teacher before starting the mindfulness programme. In the post-training phase, the questionnaire was completed by the teacher at the end of the mindfulness programme. It is noted that for each questionnaire, for each question, the possibility of not answering was given, e.g., by crossing out the space to answer, by choosing “I prefer not to answer” or “I don't know”.

## 3 Results

The data were analyzed using IBM SPSS Statistics 25 software for parametric tests, selected according to the reference sample and variables. Specifically, chi-squared test, t-test and general linear model were used.

### 3.1 Descriptive statistics and *t*-test of the pre-training questionnaire—COVID-19 effects

#### 3.1.1 Descriptive statistics

Some descriptive analyses were carried out to test whether there were significant differences between the experimental and control groups in the initial stage of the research. The results showed that there were no differences between the experimental group and the control group in the initial phase, except for the children's reactions to distance learning. Regarding distance learning, the significant difference (t = 20.255; df = 11; *p* = 0.042) between the experimental and control groups is due to the fact that the children in the experimental group showed either positive (happiness, calmness, curiosity, etc.) or negative (sadness, anger, fear, tiredness, etc.) emotions toward it, while those in the control group also reported other emotions (e.g., need for attention, boredom, hesitation, etc.). At the time of the data collection, the distance learning was no longer available for the kindergarteners, and the data collected covered a period prior to the mindfulness training. Therefore, considering the other findings, it was not considered worthwhile to investigate beyond this single result. In fact, no significant differences emerged for the other variables: children's gender, family composition during the COVID-19 period, children's direct contact with positive people, any quarantines, school closures, and new fears manifested.

#### 3.1.2 T-test analysis

The results show a better perception of the experimental group, which, despite being penalized by the repeated school closures, showed greater satisfaction with both school attendance and distance learning. They also perceived school as a safe place both before and during the pandemic period. In comparison, the control group showed a more negative reaction in terms of greater irritability, greater attachment to parents, nervousness, fear of relationships, and worry (Table 2). However, there were no significant differences between these two groups in variables related to resentment of school closing, return to school, interest in school activities, perceiving home as safe, adherence to sleep schedules, loss of appetite, questions about epidemic situation, loss of concentration, and activities during lockdown.

**Table 2:** Significant COVID effects in the pre-training questionnaire.

**Variable**	**Group**	**M**	**SD**	**t_42_**	***p***
School closure times	Experimental	2.68	0.477 0.000	6.708	<0.001
	Control	2.00			
Happy school attendance	Experimental	4.36	1.177 1.129	3.007	0.004
	Control	3.32			
Happy DL	Experimental	3.00	1.234 0.790	4.365	<0.001
	Control	1.64			
School safe place	Experimental	4.50	0.598 1.371	3.135	0.003
	Control	3.50			
Greater parental attachment	Experimental	2.23	1.412 0.868	−2.444	0.019
	Control	3.09			
Shows increased irritability	Experimental	1.64	1.255 1.058	−2.468	0.018
	Control	2.5			
Shows nervous attitudes	Experimental	1.64	1.255 1.019	−4.219	<0.001
	Control	3.09			
Show concerns	Experimental	1.50	0.964 0.790	−3.252	0.002
	Control	2.36			
Fear to relate	Experimental	1.23	0.685 0.767	−2.28	0.028
	Control	1.73			
Safe schooling	Experimental	4.23	0.813 0.727	2.543	0.015
	Control	3.64			

### 3.2 General linear model pre/post parent questionnaire—Mindfulness

To analyze the pre- and post-training questionnaires—mindfulness, a general linear model was used by means of a factor analysis between the experimental and control groups for each variable. The variables that were found to be significant are curiosity, empathy, control of emotional reaction to surprise, emotional reaction control when he/she is the center of attention, positive resolution of conflict, emotion recognition, determination, and listening to instructions (Table 3). All these variables increased significantly in the experimental group and decreased in the control group. The only exceptions were irritability and nervousness. Unlike the other variables, these tended to decrease in the experimental group and increase in the control group.

**Table 3:** Significant effects between the pre/post questionnaires—mindfulness.

**Variable**	**Group**	**Phase pre**		**phase post**		**df**	**F**	***p***
		**M**	**SD**	**M**	**SD**			
Irritability	Experimental	1.63	0.831	1.42	0.607	1,37	4.917	0.033
	Control	2.20	0.951	2.40	0.995			
Nervousness	Experimental	1.84	0.898	1.47	0.612	1,37	7.739	0.008
	Control	2.45	1.050	2.55	0.945			
Curiosity	Experimental	3.53	1.349	4.16	1.214	1,37	6.601	0.014
	Control	4.05	1.050	4.00	0.918			
Empathy	Experimental	4.26	0.562	4.42	0.607	1,37	5.837	0.021
	Control	2.60	0.821	2.35	0.745			
Control of emotional reaction to surprise	Experimental	3.00	0.882	3.21	1.032	1,37	6.079	0.018
	Control	3.50	0.513	3.35	0.587			
Emotional reaction control when he/she is the center of attention	Experimental	3.16	0.898	3.74	0.733	1,37	9.152	0.004
	Control	3.05	1.099	3.00	0.973			
Resolves conflicts in a positive way	Experimental	2.95	0.848	3.42	0,769	1,37	7.271	0.01
	Control	2.65	0.875	2.50	0.827			
Emotion recognition	Experimental	3,53	1.020	4.00	0.816	1,37	7.175	0.011
	Control	3.55	0.826	3.45	0.826			
Determination	Experimental	3.42	0.902	3,74	0.933	1,37	7.279	0.010
	Control	3.55	0.887	3.30	1.031			
Listening to instructions	Experimental	2.53	1.020	3.53	0.964	1,37	13.800	<0.001
	Control	3.05	0.887	2.90	0.852			

The variables that were not found to be significant were: worry and negative thoughts, irregular appetite, restless sleep, overreaction to anxiety, ability to pay attention, creativity and imagination, emotional response to anger, sadness, happiness, guilt, positive involvement in relationships, and recognition of mistakes.

### 3.3 General linear model pre/post SAI questionnaire

As will be described, the items were divided into three areas: cognitive, expressive, and behavioral/awareness. The general linear model was used. It was found that there was a significant increase in score in the experimental group in the cognitive area, which includes factors relating to attention, memory, spatial cognition, and reasoning; in the expressive area, which includes factors relating to creativity, language, emotions, and interpersonal relationships; and in the behavior/awareness area, which includes factors related to motor skills, awareness, planning, and control of behavior. In all these areas scores of the control group remained unchanged (Table 4).

**Table 4:** Significant variables Pre/Post SAI questionnaire.

**Variable**	**Group**	**Phase pre**		**Phase post**		**df**	**F**	***p***
		**M**	**SD**	**M**	**SD**			
Cognitive	Experimental	23.9545	5.44651	25.6818	5.09329	1,44	20.822	0.000
	Control	26.9167	2.1451	26.9167	2.1451			
Expressive	Experimental	21.2727	4.24468	23.6364	3.65859	1,44	19.998	0.000
	Control	25.5833	2.01983	25.5833	2.01983			
Behavior/Awareness	Experimental	21.1818	5.89299	23.1818	4.73725	1,44	11.223	0.002
	Control	26.5833	2.70131	26.5833	2.70131			

## 4 Discussion

When the emotional impact of COVID-19 on the children was examined, significant differences were found between the experimental group, who took part in the mindfulness programme, and the control group. In fact, the levels of irritability and nervousness decreased significantly in the experimental group, confirming that a mindfulness-based programme can be a protective factor in cases of acute stress, allowing a reduction in stress disorders (Montano and Villani, 2018; Querstret et al., 2020), anxiety (Evans et al., 2008; Lee and Semple, 2019) and depression (Segal et al., 2002), helping them to stay with their fears and uncertainties.

Protective factors and individual resources increased: curiosity and empathy, particularly in the management and recognition of both negative and positive emotions. This again confirms the potential of mindfulness (Guendelman et al., 2017; Weis et al., 2021). Although not statistically significant, the comparison between the pre- and post- training questionnaires showed positive and encouraging results in the experimental group for some variables, confirming the findings of several studies (Kabat-Zinn, 1994; Montano and Villani, 2018; Behan, 2020). In particular, worry decreased in the experimental group while it remained unchanged in the control group, as levels of creativity, imagination, and appetite did. The control of emotional states such as worry, guilt, and fear reactions also tended to improve in the experimental group. This contrasted with the control group, where levels of these variables tended to decrease. Furthermore, a trend toward significance was found for the variable “restless sleep” (F = 3.709; df = 1,37; *p* = 0.062). From this it can be concluded that sleep quality improved in the experimental group compared to the control group, confirming the claim that mindfulness training adapted to children can improve sleep quality (Huppert and Johnson, 2010). Finally, the analysis of the SAI questionnaires showed a general improvement in cognitive, expressive, and behavioral/awareness areas after the mindfulness training, confirming that participation in the programme is beneficial for factors such as attention and reasoning (Caldiroli et al., 2020; Milaré et al., 2021), creativity (Capurso et al., 2014; Henriksen et al., 2020), awareness (Weis et al., 2021), and behavioral control (Clark, 2020). We can therefore argue that by improving the dimension of awareness the participants in this pilot study were able to develop and enhance their ability to make sense of experiences and to re-read them from a different perspective, in this case in a functional and constructive way (Castiglioni and Faccio, 2010; Castiglioni and Gaj, 2020).

Mindfulness can bring great benefits to people's mental and physical health, even from a preventive perspective. Adapting mindfulness programmes for children and adolescents and promoting them in schools can help prevent the consequences of unexpected stressful events like it was the pandemic. As a matter of fact, ignoring psycho-physical wellbeing can leave people in a precarious state and even worsen their state of health, especially at critical moments that can lead to new worries and uncertainties, and thus psychological consequences such as frustration, lack of meaning, loneliness, stress, anxiety, depression, and psychosis (Shigemura et al., 2020; Castiglioni and Gaj, 2020; Venuleo et al., 2022; Gennaro et al., 2023). In fact, caring for yourself and cultivating positive emotions can help cope with uncomfortable situations and improve health.

Furthermore, from the perspective of positive psychology, mindfulness has worked on the two aspects of hedonic (Kahneman et al., 1999) and eudaimonic (Zambianchi, 2015) wellbeing by improving wellbeing and capacities at both personal and collective levels (Neff and Dahm, 2015).

## 5 Conclusion

After the programme, it was noted that the mindfulness protocol “Il fiore dentro” (Montano and Villani, 2018), applied to the children in the experimental group, confirmed our hypotheses, favoring an improvement in the cognitive, expressive, and behavioral/awareness areas, reducing the levels of irritability and nervousness that an emergency situation can provoke, and improving the recognition of emotions and empathy. The control group, on the other hand, experienced a decrease in these aspects.

However, it should be borne in mind that this was a pilot study with limitations. Firstly, the size of the sample allowed us to carry out preliminary analyses, which were nevertheless promising. Secondly, the data collection was *ad hoc* and not by means of standardized questionnaires. This was intentional, as this first phase is part of a larger project and in this first step we wanted to look at the questions in the tests to see if mindfulness training structured in this way could have an effect. The next step will be to expand the sample, use standardized tests, and validate the tests already used at this phase.

In conclusion, teaching mindfulness means improving the quality of life, enhancing psycho-physical wellbeing and increasing resilience. The school is the context par excellence that must prepare individuals for life and promote health: incorporating mindfulness into this and other educational contexts would allow one to look to the future with values such as awareness and confidence.

## Data Availability

The raw data supporting the conclusions of this article will be made available by the authors, without undue reservation.
